# Supplementary material for: Allosteric mechanism for KCNE1 modulation of KCNQ1 potassium channel activation
Source: eLife. 2020 Oct 23;9:e57680. doi: 10.7554/eLife.57680 (PMC7584456; doi:10.7554/eLife.57680)
Supplement: Supplementary file 1. — Table 2: Distance restraints used in generation of the closed state KCNQ1-KCNE1 docking model. Table 3: Distance restraints used in generation of the open state KCNQ1-KCNE1 docking model. Table 4: MolProbity statistics for KCNQ1-KCNE1 Rosetta models. Table 5: KCNQ1-KCNE1 residue contacts observed in MD simulations of the KCNQ1-KCNE1 RC and AO channel models. Table 6: Biophysical properties of IKs channels formed with KCNQ1 or KCNE1 mutants. [file elife-57680-supp1.docx]

**Supplementary File 1**

**Supplementary File 1–Table 1:** Parameters from Boltzmann function fits of normalized activation curves of KCNQ1-KCNE1 WT and cysteine mutants which were used in crosslinking experiments.

|  |  |  | **Control** | | **DTT** | | **Cu-phenanthroline** | |
| --- | --- | --- | --- | --- | --- | --- | --- | --- |
| **KCNQ1** | **KCNE1** | **N^a^** | **V_1/2app_**  **(mV)^b^** | **Slope k^b^** | **V_1/2app_**  **(mV)** | **Slope k** | **V_1/2app_**  **(mV)** | **Slope k** |
| WT | - | 9 / 5 / 5 | -9.9 ± 0.9 | 13.1 ± 0.7 | -14.4 ± 0.8 | 12.7 ± 0.7 | -12.3 ± 1.3 | 11.8 ± 1.0 |
| V141C | - | 13 / 7 / 5 | -10.5 ± 0.6 | 14.6 ± 0.5 | -13.7 ± 0.7 | 13.5 ± 0.6 | -5.1 ± 0.5 | 13.9 ± 0.4 |
| I274C | - | 12 / 7 / 7 | -21.2 ± 1.9 | 17.7 ± 1.4 | -26.6 ± 1.8 | 11.2 ± 1.5 | -31.3 ± 1.9 | 12.5 ± 1.5 |
| WT | WT | 10 / 4 / 6 | 27.7 ± 1.0 | 13.8 ± 0.9 | 31.3 ± 0.9 | 12.3 ± 0.8 | 24.9 ± 0.9 | 14.2 ± 0.8 |
| V141C | WT | 18 / 12 / 9 | 28.4 ± 1.0 | 13.6 ± 0.9 | 30.1 ± 0.9 | 12.6 ± 0.8 | 30.0 ± 1.0 | 13.1 ± 0.9 |
| I274C | WT | 20 / 9 / 11 | 24.8 ± 1.1 | 15.4 ± 1.0 | 21.6 ± 1.0 | 15.4 ± 0.9 | 24.9 ± 1.2 | 15.3 ± 1.1 |
| I274C | L45C | 6 / 6 / 5 | 28.9 ± 1.2 | 14.5 ± 1.1 | 24.9 ± 1.0 | 14.9 ± 0.9 | 25.2 ± 1.1 | 14.7 ± 1.0 |
| V141C | V47C | 8 / 6 / 6 | 12.7 ± 1.2 | 18.7 ± 1.1 | 17.0 ± 1.0 | 16.9 ± 0.9 | 16.2 ± 1.2 | 17.9 ± 1.0 |
| I274C | V47C | 13 / 10 / 7 | 25.3 ± 1.1 | 15.1 ± 1.0 | 20.5 ± 1.0 | 15.7 ± 0.9 | 23.8 ± 1.1 | 15.5 ± 1.0 |
| V141C | L48C | 7 / 5 / 5 | 10.4 ± 0.9 | 18.0 ± 0.8 | 10.9 ± 0.9 | 17.8 ± 0.8 | 6.9 ± 0.9 | 18.7 ± 0.8 |
| I274C | L48C | 12 / 7 / 7 | -11.1 ± 1.2 | 22.6 ± 0.8 | -12.0 ± 1.0 | 22.6 ± 0.7 | -13.0 ± 1.1 | 22.0 ± 0.7 |

^a^ N refers to the number of cells that were treated with control solution, DTT, or Cu-phenanthroline, respectively.

^b^ The V_1/2app_ of activation and the slope factor k were obtained by least-squares fitting with a Boltzmann function: *I*_tail_ / *I*_tail_max = (1 - *I*_Bottom_) / (1+exp[(*V*_1/2app_ - *V*)/*k*]) + *I*_Bottom_

**Supplementary File 1–Table 2:** Distance restraints used in generation of the closed state KCNQ1-KCNE1 docking model.

| **KCNQ1 Residue** | **KCNE1 Residue** | **Analysis Method** | **Reference** | **Restrained Cα-Cα Distance (Å)** | **Distance (Å)**  **Rosetta Model** | **Mean (Min – Max) Distance (Å) MD Sim.** |
| --- | --- | --- | --- | --- | --- | --- |
| ***Restraints from disulfide crosslinking experiments*** | | | | | | |
| V141* | E43* | Disulfide crosslinking | (Chan et al. 2012) | 12.0 | 10.6 | 14.3 ( 10.7 - 17.4)  12.2 ( 8.9 - 16.4)  11.1 ( 7.6 - 15.0)  9.8 ( 5.6 - 16.5) |
| V141 | A44 | Disulfide crosslinking | (Chan et al. 2012) | 12.0 | 11.6 | 14.0 ( 10.2 - 16.1)  12.4 ( 9.0 - 15.8)  10.2 ( 5.5 - 15.3)  10.5 ( 5.8 - 14.2) |
| S143* | G40* | Disulfide crosslinking | (Chung et al. 2009) | 12.0 | 11.6 | 20.4 ( 11.0 - 26.5)  17.1 ( 12.1 - 20.9)  17.9 ( 14.2 - 22.3)  14.5 ( 6.2 - 23.7) |
| T144* | G40* | Disulfide crosslinking | (Chung et al. 2009) | 12.0 | 14.5 | 18.9 ( 9.2 - 24.8)  15.4 ( 8.5 - 19.6)  16.5 ( 12.4 - 20.2)  12.7 ( 3.4 - 22.0) |
| T144 | K41 | Disulfide crosslinking | (Wang et al. 2011) | 12.0 | 13.3 | 17.3 ( 10.2 - 21.6)  12.9 ( 8.9 - 17.1)  14.2 ( 9.5 - 18.7)  12.3 ( 4.3 - 20.3) |
| T144* | E43* | Disulfide crosslinking | (Chung et al. 2009) | 12.0 | 11.7 | 13.9 ( 9.6 - 18.4)  12.8 ( 7.4 - 18.9)  12.9 ( 8.2 - 18.3)  10.9 ( 5.6 - 17.5) |
| I145* | G40* | Disulfide crosslinking | (Chung et al. 2009)  (Wang et al. 2011) | 12.0 | 11.3 | 15.3 ( 5.9 - 21.2)  12.4 ( 7.2 - 17.2)  13.8 ( 10.0 - 18.1)  11.6 ( 4.1 - 18.6) |
| I145 | K41 | Disulfide crosslinking | (Chung et al. 2009)  (Wang et al. 2011) | 12.0 | 10.6 | 13.9 ( 7.6 - 17.9)  10.0 ( 6.1 - 14.2)  11.9 ( 7.0 - 16.3)  10.5 ( 4.0 - 17.2) |
| I145 | L42 | Disulfide crosslinking | (Chung et al. 2009) | 12.0 | 7.0 | 12.0 ( 6.5 - 18.4)  8.0 ( 5.1 - 13.0)  9.4 ( 5.5 - 13.6)  8.1 ( 4.0 - 14.2) |
| I145* | E43* | Disulfide crosslinking | (Chung et al. 2009) | 12.0 | 8.6 | 10.6 ( 6.4 - 15.4)  9.9 ( 4.7 - 16.8)  11.0 ( 6.0 - 16.8)  8.7 ( 4.1 - 16.3) |
| E146* | G40* | Disulfide crosslinking | (Chung et al. 2009) | 12.0 | 12.8 | 15.0 ( 4.1 - 21.6)  13.3 ( 6.2 - 20.8)  15.8 ( 11.2 - 21.0)  13.1 ( 4.2 - 20.6) |
| E146* | K41* | Disulfide crosslinking | (Chung et al. 2009) | 12.0 | 13.1 | 14.0 ( 5.7 - 19.4)  11.4 ( 7.7 - 17.1)  14.0 ( 8.5 - 19.3)  12.2 ( 3.9 - 19.8) |
| Q147 | S37 | Disulfide crosslinking | (Wang et al. 2011) | 12.0 | 9.4 | 16.5 ( 3.6 - 28.8)  14.0 ( 4.5 - 23.5)  16.3 ( 5.1 - 26.7)  13.2 ( 5.3 - 25.6) |
|  |  |  |  |  |  |  |
| W323* | E43* | Disulfide crosslinking | (Chung et al. 2009) | 12.0 | 10.2 | 5.5 ( 4.3 - 8.8)  9.1 ( 6.3 - 13.1)  8.9 ( 5.3 - 14.2)  9.2 ( 5.2 - 14.5) |
| V324 | K41 | Disulfide crosslinking | (Chung et al. 2009) | 12.0 | 7.6 | 8.7 ( 6.4 - 16.1)  10.6 ( 6.4 - 17.3)  9.4 ( 7.0 - 16.2)  14.0 ( 7.5 - 22.3) |
| ***Restraints from additional functional experiments*** | | | | | | |
| I274 | L45 | Higher I_Ks_ of double Cys mutant under reducing cond (+DTT) | This study | 12.0 | 15.5 | 16.5 ( 14.6 - 19.2)  15.9 ( 13.8 - 20.2)  15.9 ( 13.7 - 18.1)  15.8 ( 13.5 - 18.7) |
| I328 | L51 | Lower I_Ks_ of double Cys mutant when Cd(II) present  Cd(II)-cysteine crosslinking | (Tapper and George 2001) | 15.0 | 15.3 | 15.2 ( 12.6 - 17.6)  15.2 ( 11.9 - 18.1)  15.3 ( 12.9 - 17.7)  14.7 ( 11.6 - 18.1) |
| C331 | F54 | Lower I_Ks_ of Q1 with E1 F54C mutant when Cd(II) present  Cd(II)-cysteine crosslinking | (Tapper and George 2001) | 15.0 | 13.4 | 12.5 ( 8.9 - 14.7)  12.3 ( 8.9 - 14.9)  12.3 ( 8.7 - 16.3)  11.2 ( 7.8 - 16.0) |
| C331 | G55 | Lower I_Ks_ of Q1 with E1 G55C mutant when Cd^2+^ present  Cd(II)-cysteine crosslinking | (Tapper and George 2001) | 15.0 | 15.5 | 15.3 ( 11.4 - 17.7)  14.9 ( 12.1 - 17.6)  14.9 ( 10.6 - 17.5)  14.2 ( 10.4 - 19.0) |
| F340 | T58 | Double mutant cycle analysis | (Strutz-Seebohm et al. 2011)  (Li et al. 2014) | 12.0 | 17.4 | 17.3 ( 13.6 - 21.7)  17.5 ( 14.7 - 21.1)  17.5 ( 15.1 - 20.3)  17.8 ( 14.4 - 21.3) |
| A344 | Y65 | Double mutant cycle analysis | (Li et al. 2014) | 12.0 | 22.7 | 24.8 ( 20.1 - 30.8)  24.1 ( 20.3 - 28.7)  22.9 ( 19.6 - 27.8)  22.7 ( 19.9 - 26.6) |
| *For those restraints, the functional channel state under the experimental conditions was unclear. They were included in the restraint list of both the open and closed state model. | | | | | | |

**Supplementary File 1–Table 3:** Distance restraints used in generation of the open state KCNQ1-KCNE1 docking model.

| **KCNQ1 Residue** | **KCNE1 Residue** | **Analysis Method** | **Reference** | **Restrained Cα-Cα Distance (Å)** | **Distance (Å)**  **Rosetta Model** | **Mean (Min – Max) Distance (Å) MD Sim.** |
| --- | --- | --- | --- | --- | --- | --- |
| ***Restraints from disulfide crosslinking experiments*** | | | | | | |
| V141* | E43* | Disulfide crosslinking | (Chan et al. 2012) | 12.0 | 8.5 | 10.4 ( 7.2 - 14.0)  11.7 ( 8.7 - 14.9)  11.7 ( 8.3 - 16.6)  12.1 ( 7.3 - 16.6) |
| V141 | A44 | Disulfide crosslinking | (Wang et al. 2011)  (Chan et al. 2012) | 12.0 | 6.3 | 8.7 ( 5.9 - 11.7)  9.1 ( 6.5 - 11.7)  8.4 ( 6.0 - 13.5)  9.7 ( 6.2 - 13.5) |
| S143* | G40* | Disulfide crosslinking | (Chung et al. 2009) | 12.0 | 11.4 | 11.6 ( 8.4 - 17.7)  13.4 ( 9.8 - 20.3)  13.6 ( 10.0 - 19.8)  13.5 ( 8.8 - 19.9) |
| T144 | G40 | Disulfide crosslinking | (Chung et al. 2009)  (Wang et al. 2011) | 12.0 | 10.5 | 10.6 ( 7.1 - 16.9)  12.4 ( 7.4 - 19.6)  11.3 ( 7.4 - 19.3)  12.4 ( 8.2 - 19.3) |
| T144* | K41* | Disulfide crosslinking | (Chung et al. 2009) | 12.0 | 11.8 | 12.8 ( 9.0 - 16.2)  13.5 ( 9.4 - 17.9)  10.7 ( 7.0 - 19.1)  13.8 ( 9.9 - 19.1) |
| T144* | E43* | Disulfide crosslinking | (Chung et al. 2009) | 12.0 | 9.6 | 11.1 ( 8.4 - 15.2)  12.2 ( 8.6 - 16.2)  12.3 ( 8.9 - 18.9)  13.2 ( 8.7 - 18.9) |
| I145 | G40 | Disulfide crosslinking | (Chung et al. 2009)  (Wang et al. 2011) | 12.0 | 6.9 | 7.1 ( 4.9 - 13.1)  8.8 ( 5.1 - 15.9)  8.8 ( 5.2 - 15.6)  8.7 ( 5.0 - 15.6) |
| I145 | K41 | Disulfide crosslinking | (Chung et al. 2009)  (Wang et al. 2011) | 12.0 | 8.6 | 9.5 ( 6.2 - 13.2)  10.1 ( 6.5 - 14.8)  7.8 ( 4.9 - 15.4)  10.4 ( 6.1 - 15.4) |
| I145 | L42 | Disulfide crosslinking | (Chung et al. 2009) | 12.0 | 10.2 | 11.2 ( 9.3 - 14.9)  12.0 ( 9.3 - 15.5)  10.5 ( 6.6 - 18.0)  12.5 ( 8.6 - 18.0) |
| I145* | E43* | Disulfide crosslinking | (Chung et al. 2009) | 12.0 | 7.4 | 8.6 ( 6.5 - 13.1)  9.4 ( 6.0 - 14.0)  10.5 ( 6.5 - 16.3)  10.3 ( 6.3 - 16.3) |
| E146* | G40* | Disulfide crosslinking | (Chung et al. 2009) | 12.0 | 8.1 | 8.5 ( 5.2 - 14.8)  9.6 ( 6.2 - 17.0)  10.7 ( 4.8 - 17.3)  9.7 ( 4.8 - 17.3) |
| E146* | K41* | Disulfide crosslinking | (Chung et al. 2009) | 12.0 | 10.5 | 11.1 ( 7.5 - 15.4)  11.5 ( 7.8 - 15.9)  9.9 ( 6.7 - 17.2)  11.7 ( 7.1 - 17.2) |
| Q147 | G38 | Disulfide crosslinking | (Wang et al. 2011) | 12.0 | 10.9 | 10.0 ( 3.6 - 16.9)  9.8 ( 4.6 - 18.8)  13.7 ( 5.2 - 21.3)  11.7 ( 4.0 - 21.3) |
| Q147 | G40 | Disulfide crosslinking | (Wang et al. 2011) | 12.0 | 6.7 | 6.9 ( 4.4 - 13.0)  7.7 ( 4.6 - 15.4)  10.8 ( 4.6 - 15.8)  7.9 ( 4.6 - 15.6) |
| Q147 | K41 | Disulfide crosslinking | (Wang et al. 2011) | 12.0 | 8.4 | 8.7 ( 5.0 - 13.9)  9.0 ( 5.6 - 13.5)  9.2 ( 5.6 - 15.4)  9.1 ( 5.5 - 15.4) |
| W323* | E43* | Disulfide crosslinking | (Chung et al. 2009) | 12.0 | 10.5 | 10.8 ( 7.4 - 14.1)  9.9 ( 6.0 - 15.9)  8.6 ( 5.1 - 17.1)  10.9 ( 5.0 - 17.1) |
| V324 | L42 | Disulfide crosslinking | (Chung et al. 2009) | 12.0 | 12.8 | 13.5 ( 9.6 - 17.6)  12.8 ( 9.8 - 19.9)  14.0 ( 10.5 - 20.7)  14.8 ( 9.9 - 20.7) |
| ***Restraints from additional functional experiments*** | | | | | | |
| V141 | L48 | Lower I_Ks_ of double Cys mutant under reducing cond. (+DTT) | This study | 12.0 | 8.6 | 9.9 ( 8.2 - 12.4)  9.0 ( 6.1 - 12.9)  8.8 ( 5.7 - 15.0)  10.6 ( 7.9 - 15.0) |
| C331 | F54 | Positive shift in V_1/2_ of Q1 with E1 F54C mutant under reducing cond. (+DTT).  Constitutive current under oxidizing cond. (+H_2_O_2_) that is reversed by H_2_O_2_ washout and DTT. | (Wang et al. 2012) | 12.0 | 13.0 | 10.8 ( 8.1 - 13.6)  13.6 ( 9.5 - 16.7)  12.8 ( 9.5 - 17.9)  13.3 ( 9.5 - 17.9) |
| *For those restraints, the functional channel state under the experimental conditions was unclear. They were included in the restraint list of both the open and closed state model. | | | | | | |

**Supplementary File 1–Table 4:** MolProbity statistics for the KCNQ1-KCNE1 Rosetta models.

|  | **Open state model** | **Closed state model** |
| --- | --- | --- |
| Molprobity score | 1.34 (98^th^ percentile) | 1.35 (98^th^ percentile) |
| Clash score | 2.36 (99^th^ percentile) | 2.39 (99^th^ percentile) |
| Ramachandran statistic |  |  |
| Favored regions (%) | 95.3 | 95.2 |
| Allowed regions (%) | 4.5 | 3.9 |
| Disallowed regions (%) | 0.2 | 0.9 |
| Rotamer statistic |  |  |
| Favored rotamers (%) | 99.5 | 99.5 |
| Poor rotamers (%) | 0.0 | 0.0 |
| Cβ deviations (%) | 0.0 | 0.1 |
| Bad bonds (%) | 0.0 | 0.0 |
| Bad angles (%) | 0.06 | 0.04 |
| Rosetta ΔG_Binding_ (REU)^a^ | -63.3 ± 1.3 | -64.7 ± 1.8 |
| Amber ΔG_Binding_ (kcal/mol)^a^ | -59.6 ± 1.5 | -59.2 ± 1.0 |

^a^ mean ± SEM

**Supplementary File 1–Table 5:** KCNQ1-KCNE1 residue contacts observed in MD simulations of the KCNQ1-KCNE1 RC and AO channel models. For every KCNE1 residue, the KCNQ1 residues that made more than one heteroatom contact on average (≤ 4 Å distance) are listed. Residue numbers are colored differently to indicate different KCNQ1 subunits. The average contact number is given in parentheses.

| **KCNE1 Residue** | **Interacting KCNQ1 Residue – RC Model** | **Interacting KCNQ1 Residue – AO Model** |
| --- | --- | --- |
| S37 | **E290** (1.3) | **Q147** (1.0), **G297** (1.9), **D301** (2.0) |
| G38 | **Q321** (1.1), **W323** (1.2) | **Q147** (1.3) |
| D39 | **Q321** (2.2), **T322** (2.0), **W323** (2.4) | **Q147** (2.4) |
| G40 | **W323** (3.4) | **I145** (1.7), **Q147** (2.4), **Y148** (2.3) |
| K41 | **S298** (1.2), **D301** (1.8), **Q321** (1.8), **W323** (6.1) | **Q147** (2.3), **Y148** (5.3) |
| L42 | **I145** (2.4), **Q147** (2.1), **Y148** (1.8), **W323** (1.2) | **W323** (2.9) |
| E43 | **I145** (1.1), **Q147** (1.1), **Y148** (2.0), **W323** (2.5) | **I145** (1.8), **S298** (6.4), **Y299** (1.9), **A300** (2.7), **W323** (5.9) |
| A44 | **W323** (5.0) | **L142** (1.3), **I145** (1.2), **Y148** (1.4) |
| L45 | **W323** (4.1) | - |
| Y46 | **A300** (3.8), **L303** (4.2), **W304** (11.9), **V307** (2.0), **W323** (5.5), **K326** (2.2), **T327** (6.8), **S330** (6.1) | **A300** (3.1), **L303** (1.2), **W304** (3.3), **W323** (14.7), **V324** (2.0), **T327** (2.5) |
| V47 | **I138** (1.1), **V141** (2.4), **Y299** (2.4), **A300** (3.3), **L303** (2.3) | **V141** (1.6), **Y299** (3.7), **A300** (1.6), **L303** (1.1) |
| L48 | **I138** (3.8), **V141** (1.1) | **I138** (2.7) |
| M49 | **T327** (2.3) | - |
| V50 | **F270** (1.4), **L303** (2.6) | **F270** (1.6), **L303** (1.6) |
| L51 | **L134** (4.5), **L137** (2.0), **I138** (2.5) | **L134** (3.2), **L137** (2.5), **I138** (3.4), **I274** (1.2) |
| G52 | - | - |
| F53 | **F270** (3.0), **C331** (1.7) | **F270** (2.9) |
| F54 | **F130** (3.7), **F232** (5.8), **Y267** (6.3), **F270** (11.7), **L271** (1.6), **I274** (2.5) | **F130** (1.5), **M238** (2.1), **Y267** (6.3), **F270** (10.5), **L271** (3.0), **I274** (3.3) |
| G55 | **F127** (1.4), **F130** (2.6), **L134** (1.1) | **F130** (3.4), **L134** (1.3) |
| F56 | - | - |
| F57 | **I263** (2.6), **L266** (4.5), **Y267** (9.3), **F270** (4.4) | **I263** (2.8), **L266** (4.9), **Y267** (8.1), **F270** (4.7) |
| T58 | **F127** (2.4), **F130** (7.5), **Y267** (6.1) | **F130** (8.2), **V241** (1.2), **Y267** (8.1) |
| L59 | **F127** (7.8) | **F123** (2.0), **F127** (6.5) |
| G60 | - | - |
| I61 | **L239** (1.5), **I263** (4.5), **Y267** (4.3) | **G245** (2.7), **T247** (1.8), **Q260** (1.8), **I263** (4.3), **T264** (1.9), **Y267** (7.3) |
| M62 | **F123** (7.5), **F127** (4.7), **L239** (1.5) | **F123** (6.6), **H126** (1.6), **F127** (3.4), **F130** (1.6), **V241** (2.1), **Q244** (10.0), **G245** (1.5) |
| L63 | - | - |
| S64 | **R259** (3.4), **Q260** (2.3) | **R259** (1.7), **Q260** (7.0), **I263** (1.4) |
| Y65 | **F123** (6.5), **L239** (6.8), **H240** (2.0), **D242** (3.7), **R243** (15.1), **Q244** (5.5), **R259** (1.0), **Q260** (5.4) | **R116** (4.1), **P117** (2.8), **R243** (1.8), **Q244** (10.4), **G245** (13.4), **G246** (7.0), **T247** (1.4), **Q260** (4.2) |
| I66 | **W120** (3.1), **F123** (3.9) | **T118** (3.6), **F123** (2.9), **Q244** (3.0) |
| R67 | **F256** (1.4), **R259** (1.4) | **R259** (3.6), **Q260** (1.3) |
| S68 | **R243** (3.4), **R259** (2.6) | **R259** (1.5), **Q260** (3.1), **K362** (1.2) |
| K69 | **D242** (1.8), **R243** (2.6) | **R116** (2.9), **T118** (3.8), **R366** (1.0) |
| K70 | **W120** (1.6), **R243** (1.1) | **T118** (1.0), **R366** (1.4) |
| L71 | **R243** (1.2) | **Q359** (1.3), **Q361** (1.0), **K362** (1.8), **N365** (1.1), **R366** (1.1) |

**Supplementary File 1–Table 6:** Biophysical properties of I_Ks_ channels formed with KCNQ1 or KCNE1 mutants.

| **Q1/E1**  **Variant** | **Current**  **(%WT)** | **V_1/2app_**  **(mV)^c,e^** | **Slope k^c,e^** | **N^d^** | **Activation τ (sec)** | | | | | | | **Deactivation τ (sec)** | | | | | | | | |
| --- | --- | --- | --- | --- | --- | --- | --- | --- | --- | --- | --- | --- | --- | --- | --- | --- | --- | --- | --- | --- |
|  |  |  |  |  | 0 mV | 10 mV | 20 mV | 30 mV | 40 mV | 50 mV | 60 mV | -100 mV | -90 mV | -80 mV | -70 mV | -60 mV | -50 mV | -40 mV | -30 mV | -20 mV |
| **KCNQ1^a^** |  |  |  |  |  |  |  |  |  |  |  |  |  |  |  |  |  |  |  |  |
| WT | 100.0 | 28.6 ± 0.7 | 11.4 ± 0.2 | 77 / 45 | - | - | 9.94 | 6.92 | 5.53 | 3.84 | 3.52 | 0.11 | 0.12 | 0.12 | - | 0.27 | 0.24 | 0.25 | 0.26 | 0.28 |
| Y267F | 20.0 | **42.1 ± 0.7** | **8.5 ± 0.3** | 61 / 22 | - | - | 5.43 | 4.81 | 2.90 | 2.79 | 3.48 | - | - | - | - | - | - | - | **0.10** | **0.11** |
| W323A | 25.4 | **22.2 ± 0.7** | **15.2 ± 0.8** | 56 / 22 | 6.49 | 4.02 | 3.21 | 2.65 | **2.30** | **1.80** | **1.59** | 0.10 | - | - | - | - | - | 0.24 | 0.24 | 0.26 |
| W323L | 17.8 | **17.3 ± 1.5** | **19.0 ± 1.0** | 61 / 25 | 1.98 | 1.79 | **1.62** | **1.52** | **1.60** | **1.49** | **1.44** | **0.09** | 0.10 | - | - | - | - | 0.30 | 0.38 | 0.33 |
| W323F | 83.3 | 29.1 ± 0.5 | **12.8 ± 0.2** | 72 / 58 | 5.28 | 4.69 | 4.57 | 3.15 | **2.58** | **2.17** | **1.81** | **0.08** | **0.08** | 0.09 | - | **0.17** | **0.19** | **0.22** | 0.27 | 0.28 |
| K362A | 23.2 | **43.3 ± 0.6** | **7.8 ± 0.4** | 65 / 31 | - | - | - | - | - | 8.59 | 7.57 | **0.07** | - | - | - | - | - | **0.10** | **0.11** | **0.12** |
| N365A | 25.4 | **43.3 ± 0.6** | **7.8 ± 0.4** | 67 / 24 | - | - | 4.13 | 3.63 | 2.99 | 2.66 | 2.54 | **0.09** | **0.09** | - | - | - | - | **0.11** | **0.12** | **0.14** |
| **KCNE1^b^** |  |  |  |  |  |  |  |  |  |  |  |  |  |  |  |  |  |  |  |  |
| WT | 100.0 | 24.4 ± 0.5 | 13.6 ± 0.1 | 320 / 270 | 6.86 | 6.89 | 7.30 | 5.81 | 4.40 | 3.24 | 2.54 | 0.14 | 0.14 | 0.13 | 0.29 | 0.28 | 0.27 | 0.27 | 0.26 | 0.26 |
| Y46A | 26.2 | **-11.9 ± 5.1** | 15.1 ± 1.6 | 41 / 14 | **1.23** | **1.20** | **1.12** | **1.07** | **1.15** | **1.05** | **0.99** | 0.13 | 0.15 | 0.12 | - | - | 0.42 | 0.41 | 0.42 | 0.29 |
| Y46L | 8.5 | **-3.3 ± 3.1** | 15.8 ± 1.8 | 52 / 14 | 4.70 | **2.71** | **1.71** | **1.45** | **1.33** | **1.18** | **1.61** | 0.13 | 0.15 | 0.13 | - | - | - | - | - | - |
| Y46F | 70.6 | 23.2 ± 1.3 | 13.5 ± 0.4 | 39 / 28 | - | - | 6.87 | 5.80 | 3.47 | 2.94 | 2.19 | 0.12 | 0.12 | - | - | - | 0.24 | 0.24 | 0.24 | **0.22** |
| V50A | 41.4 | 22.0 ± 0.9 | 14.4 ± 0.4 | 74 / 43 | 6.94 | 8.20 | 7.51 | 3.72 | 4.81 | **2.16** | 1.96 | 0.14 | 0.14 | 0.20 | - | **0.36** | **0.37** | **0.38** | **0.38** | **0.38** |
| F56A | 50.3 | 24.9 ± 1.2 | 13.5 ± 0.3 | 42 / 30 | - | - | 12.40 | 7.82 | 4.84 | 3.80 | 2.38 | **0.11** | **0.12** | - | - | - | - | 0.28 | 0.23 | **0.22** |
| F57A | 11.4 | 18.6 ± 3.0 | **17.1 ± 1.0** | 47 / 14 | 6.94 | 4.12 | 4.39 | **2.79** | **2.38** | **1.54** | 2.21 | 0.19 | 0.17 | - | - | - | - | - | - | - |
| F57L | 169.7 | **6.7 ± 1.5** | **15.5 ± 0.3** | 73 / 58 | **0.90** | **0.74** | **0.64** | **0.57** | **0.70** | **0.51** | **0.46** | 0.13 | 0.13 | 0.11 | 0.31 | 0.24 | 0.24 | 0.24 | 0.25 | 0.27 |
| L59A | 48.9 | **29.6 ± 1.2** | **11.9 ± 0.5** | 46 / 29 | - | 13.30 | 8.64 | 6.39 | 4.24 | 3.36 | 2.03 | **0.10** | 0.12 | - | - | 0.22 | 0.19 | **0.19** | **0.21** | 0.22 |
| L63A | 115.8 | 25.3 ± 1.3 | 12.8 ± 0.4 | 30 / 20 | 7.36 | 6.40 | 5.69 | 5.36 | 4.75 | 2.98 | 2.57 | 0.12 | 0.12 | 0.13 | 0.23 | 0.29 | 0.26 | 0.31 | 0.34 | 0.35 |
| Y65A | 55.7 | 28.7 ± 2.7 | 12.1 ± 0.9 | 27 / 18 | 5.62 | 6.00 | **3.30** | **2.66** | **2.48** | **2.10** | 2.03 | **0.08** | **0.10** | 0.11 | - | **0.12** | 0.18 | **0.18** | **0.17** | **0.17** |
| Y65L | 12.3 | **36.7 ± 1.6** | 13.2 ± 1.5 | 66 / 23 | - | - | - | **3.02** | **1.79** | 2.50 | 2.27 | **0.09** | 0.10 | 0.11 | **0.09** | - | - | - | - | - |
| Y65F | 66.0 | **33.8 ± 0.7** | **11.2 ± 0.3** | 34 / 23 | - | - | - | 13.71 | 6.85 | 5.54 | 5.43 | 0.12 | **0.11** | - | - | 0.23 | **0.23** | **0.23** | 0.27 | 0.26 |
| I66A | 97.7 | **32.6 ± 0.6** | **11.3 ± 0.3** | 41 / 32 | 5.91 | 7.10 | 5.52 | 4.11 | 3.41 | **1.98** | **1.44** | **0.08** | **0.10** | 0.11 | - | **0.15** | **0.16** | **0.18** | **0.19** | **0.21** |

^a^ KCNQ1 variants were measured in CHO-K1 cells stably expressing human KCNE1 and transiently transfected with WT or mutant KCNQ1 cDNA (see Methods & Materials).

^b^ KCNE1 variants were measured in CHO-K1 cells co-transfected with human KCNQ1 cDNA and WT or mutant KCNE1 cDNA (see Methods & Materials).

^c^ The V_1/2app_ of activation and slope factor k were obtained by least-squares fitting with a Boltzmann function (mean ± SEM).

^d^ N refers to the number of cells used for determination of peak current / voltage-dependence of activation.

^e^ Bold text indicates variants of KCNQ1 or KCNE1 that were significantly different from WT KCNQ1 or WT KCNE1 in terms of the specified parameter (Student’s t-test, P < 0.001).

**Supplementary References**

Chan, Priscilla J., Jeremiah D. Osteen, Dazhi Xiong, Michael S. Bohnen, Darshan Doshi, Kevin J. Sampson, Steven O. Marx, Arthur Karlin, and Robert S. Kass. 2012. "Characterization of KCNQ1 atrial fibrillation mutations reveals distinct dependence on KCNE1." *J Gen Physiol* 139:135-144.

Chung, David Y., Priscilla J. Chan, John R. Bankston, Lin Yang, Guoxia Liu, Steven O. Marx, Arthur Karlin, and Robert S. Kass. 2009. "Location of KCNE1 relative to KCNQ1 in the I(KS) potassium channel by disulfide cross-linking of substituted cysteines." *Proc Natl Acad Sci U S A* 106:743-748.

Li, P., H. Liu, C. Lai, P. Sun, W. Zeng, F. Wu, L. Zhang, S. Wang, C. Tian, and J. Ding. 2014. "Differential modulations of KCNQ1 by auxiliary proteins KCNE1 and KCNE2." *Sci Rep* 4:4973. doi: 10.1038/srep04973.

Strutz-Seebohm, Nathalie, Michael Pusch, Steffen Wolf, Raphael Stoll, Daniel Tapken, Klaus Gerwert, Bernard Attali, and Guiscard Seebohm. 2011. "Structural basis of slow activation gating in the cardiac I Ks channel complex." *Cell Physiol Biochem* 27:443-452.

Tapper, A. R., and A. L. George. 2001. "Location and orientation of minK within the I(Ks) potassium channel complex." *J Biol Chem* 276:38249-38254.

Wang, Y., M. Zhang, Y. Xu, M. Jiang, D. P. Zankov, M. Cui, and G. N. Tseng. 2012. "Probing the structural basis for differential KCNQ1 modulation by KCNE1 and KCNE2." *J Gen Physiol* 140 (6):653-69. doi: 10.1085/jgp.201210847.

Wang, Yu Hong, Min Jiang, Xu Lin Xu, Kai-Ling Hsu, Mei Zhang, and Gea-Ny Tseng. 2011. "Gating-related molecular motions in the extracellular domain of the IKs channel: implications for IKs channelopathy." *J Membr Biol* 239:137-156.
